# Iron Acquisition in *Bacillus cereus*: The Roles of IlsA and Bacillibactin in Exogenous Ferritin Iron Mobilization

**DOI:** 10.1371/journal.ppat.1003935

**Published:** 2014-02-13

**Authors:** Diego Segond, Elise Abi Khalil, Christophe Buisson, Nadine Daou, Mireille Kallassy, Didier Lereclus, Paolo Arosio, Fadi Bou-Abdallah, Christina Nielsen Le Roux

**Affiliations:** 1 INRA, UMR 1319 Micalis, La Minière, Guyancourt, France; 2 AgroParisTech, UMR Micalis, Jouy en Josas, France; 3 Laboratory of Biotechnology, Saint-Joseph University, Beyrouth, Lebanon; 4 Department of Medicine, Section of Infectious Diseases, Boston University School of Medicine, Boston, Massachusetts, United States of America; 5 Department of Molecular and Translational Medicine, University of Brescia, Brescia, Italy; 6 Department of Chemistry, State University of New York at Potsdam, Potsdam, New York, United States of America; University of Tubingen, Germany

## Abstract

In host-pathogen interactions, the struggle for iron may have major consequences on the outcome of the disease. To overcome the low solubility and bio-availability of iron, bacteria have evolved multiple systems to acquire iron from various sources such as heme, hemoglobin and ferritin. The molecular basis of iron acquisition from heme and hemoglobin have been extensively studied; however, very little is known about iron acquisition from host ferritin, a 24-mer nanocage protein able to store thousands of iron atoms within its cavity. In the human opportunistic pathogen *Bacillus cereus*, a surface protein named IlsA (Iron-regulated leucine rich surface protein type A) binds heme, hemoglobin and ferritin *in vitro* and is involved in virulence. Here, we demonstrate that IlsA acts as a ferritin receptor causing ferritin aggregation on the bacterial surface. Isothermal titration calorimetry data indicate that IlsA binds several types of ferritins through direct interaction with the shell subunits. UV-vis kinetic data show a significant enhancement of iron release from ferritin in the presence of IlsA indicating for the first time that a bacterial protein might alter the stability of the ferritin iron core. Disruption of the siderophore bacillibactin production drastically reduces the ability of *B. cereus* to utilize ferritin for growth and results in attenuated bacterial virulence in insects. We propose a new model of iron acquisition in *B. cereus* that involves the binding of IlsA to host ferritin followed by siderophore assisted iron uptake. Our results highlight a possible interplay between a surface protein and a siderophore and provide new insights into host adaptation of *B. cereus* and general bacterial pathogenesis.

## Author Summary

Iron homeostasis is important for all living organisms; too much iron confers cell toxicity, and too little iron results in reduced cell fitness. While crucial for many cellular processes in both man and pathogens, a battle for this essential nutrient erupts during infection between the host and the invading bacteria. Iron is principally stored in ferritin, a large molecule able to bind several thousand iron ions. Although host ferritins represent a mine of iron for pathogens, studies of the mechanisms involved in its acquisition by bacteria are scarce. In the human opportunistic pathogen *Bacillus cereus*, the surface protein IlsA is able to bind several host iron sources *in vitro*. In this study, we show that IlsA acts as a ferritin receptor and enhances iron release from the ferritin through direct interaction with each ferritin subunit. Moreover, we demonstrate that the siderophore bacillibactin, a small secreted iron chelator, is essential for ferritin iron acquisition and takes part in *B. cereus* virulence. We propose a new iron acquisition model that provides new insights into bacterial host adaptation.

## Introduction

Iron is an essential nutrient for most forms of life. Owing to the high variability of the Fe^3+^/Fe^2+^ redox potential, this transition metal fulfills a large number of biological processes including respiration and DNA synthesis. However, because of its low solubility and propensity to generate highly reactive hydroxyl radicals, iron is a double-edged element and its homeostasis must be finely tuned [1]. Given that most microorganisms require micromolar iron concentrations for growth and multiplication [2], the ability to obtain iron is thus an important adaptation factor requiring intricately sophisticated iron uptake systems [3]. Upon infection, the host sets up a form of nutritional immunity aimed at depriving the invader of nutritional iron through iron redistribution in the organism and scavenging of certain microbial siderophores [4], [5]. The importance of this strategy is evidenced by the effects of iron homeostasis disorders on both innate and acquired immune responses [2], [6]. In this battle for iron, and to circumvent host-iron withholding, pathogenic bacteria are able to acquire iron via siderophore-based systems or by surface and membrane anchored proteins which interfere with host iron-containing proteins such as transferrins, hemoproteins or ferritins [7]. Most of these iron acquisition systems are under the control of the global regulator Fur (Ferric uptake regulator) which also regulates the expression of multiple virulence factors [8]. Over the past 10 years, our understanding of iron import into bacteria has been greatly improved [9], [10]. The most impressive advances concerned heme acquisition in Gram-positive bacteria. One major discovery has been made with the characterization of the Isd (Iron-regulated surface determinant) system in *Staphylococcus aureus* [11]. Heme or hemoglobin interaction with this system relies on cell wall–anchored proteins that act as hemoprotein receptors by means of their NEAT (NEAr iron Transporter) domains. Since then, a growing number of studies have emphasized the role of NEAT domains in heme binding in several Gram-positive bacteria including *S. aureus*, *Streptococcus pyogenes*, *Bacillus anthracis* (for review, see [12]) or *Bacillus cereus* (Abi Khalil *et al.*, unpublished data). Although most iron is bound to hemoglobin in vertebrates, ferritin can represent another important source of iron for microbes [13], [14], [15], [16], [17], [18], [19].

Ferritin is a well-studied ubiquitous protein found in both prokaryotes and eukaryotes. In animals, it is composed of 24 subunits that self-assemble through non-covalent interactions into a hollow spherical shell. Due to the presence of two types of subunits, H (heavy) and L (light), multiple ferritin isoforms exist whereby up to 4500 iron atoms can be mineralized inside the nanocage [20]. Whereas the main role of ferritin is to store iron under a safe and bioavailable form, other biological functions have been proposed [21]. This extremely stable protein represents a concentrated source of iron and thus could be a perfect target for microbes. However, the molecular basis of ferritin utilization by pathogens remains poorly documented. Recent studies have shown that the intracellular bacterium *Neisseria meningitidis* can indirectly utilize ferritin by inducing an iron starvation state within epithelial cells leading to ferritin degradation and release of free iron [17]. Other *in vitro* studies showed that ferritin utilization relies on proteolytic degradation in the cystic fibrosis-associated pathogen *Burkholderia cenocepacia* [19] and on a reductase activity in *Listeria monocytogenes* [22]. To our knowledge, only two molecular determinants directly involved in iron acquisition from ferritin have been identified: (i) Als3, a *Candida albicans* invasin-like protein [13] and (ii) IlsA, a *Bacillus cereus* surface protein [14]. It has been suggested that both proteins are ferritin receptors but direct *in vitro* binding to ferritin has only been reported in *B. cereus* [14].

*B. cereus* is a Gram-positive, spore-forming bacterium. This opportunistic human pathogen is frequently associated with food-borne infections due to the production of diarrheal and emetic toxins. Rare non-gastrointestinal infections such as meningitis, pneumonia, endophthalmitis or gas gangrene-like cutaneous infections have also been observed [23]. As a member of the *B. cereus sensu lato* group, *B. cereus* is closely related to other species of this group such as *B. anthracis*, the etiological agent of anthrax in mammals and the entomopathogen, *Bacillus thuringiensis* [24]. The ability of *B. cereus* and *B. thuringiensis* to colonize the host (mammal or insect) is linked to the presence of multiple adaptation and virulence factors, one of which is the capacity to acquire iron [25]. Several host iron sources can be used by *B. cereus*, including hemoproteins and ferritin [14], [26], [27], [28], [29], [30], [31]. In the past few years, various systems involved in iron acquisition have been discovered and some of them are required for full virulence in animal models (Table S1). The adaptation of *B. cereus* to iron paucity in host tissues is also illustrated by the Fur-regulation of the cytotoxin HlyII [32]. Among the iron uptake systems characterized in *B. cereus*, a surface protein, IlsA (NCBI gene number Bc1331), is involved in both ferritin and heme/hemoglobin acquisition [14], [33]. This protein is composed of a unique combination of three conserved domains: an N-Terminal NEAT domain followed by 13 LRRs (Leucine Rich Repeat) and three C-Terminal SLH (S-Layer Homology) domains and has been shown to interact with heme and hemoglobin via the NEAT domain [14] (Abi Khalil *et al.*, unpublished data, a revised manuscript has been re-submitted to Metallomics). Affinity tests revealed that IlsA binds to ferritin although the details of this interaction have not been described [14]. As a Fur-regulated gene, *ilsA* is specifically expressed in the insect hemocoel and under iron-depleted conditions [14], [33]. Moreover, the virulence and the survival of the *ΔilsA* mutant are reduced in the lepidopteran insect model *Galleria mellonella* suggesting IlsA involvement in optimal colonization of a susceptible host [14].

These results prompted us to investigate the interaction between IlsA and ferritin and examine the possible involvement of two siderophores produced by *B. cereus*, bacillibactin and petrobactin, as partners of IlsA in ferritin utilization. Here, we demonstrate that IlsA acts as ferritin receptor on the surface of *B. cereus*. Isothermal titration calorimetry data indicate a binding stoichiometry of 24 IlsA per ferritin molecule (i.e. one IlsA per ferritin subunit). *In vitro* iron release kinetics showed significant increase of iron mobilization from ferritin in the presence of IlsA. In addition, our *in vivo* results show that bacillibactin is essential for iron acquisition from ferritin and for full virulence of *B. cereus* in an insect model, suggesting that IlsA and bacillibactin may work synergistically to effectively mobilize iron from host ferritin.

## Results

### IlsA is required for ferritin binding *in vivo*

**Figure 1:**
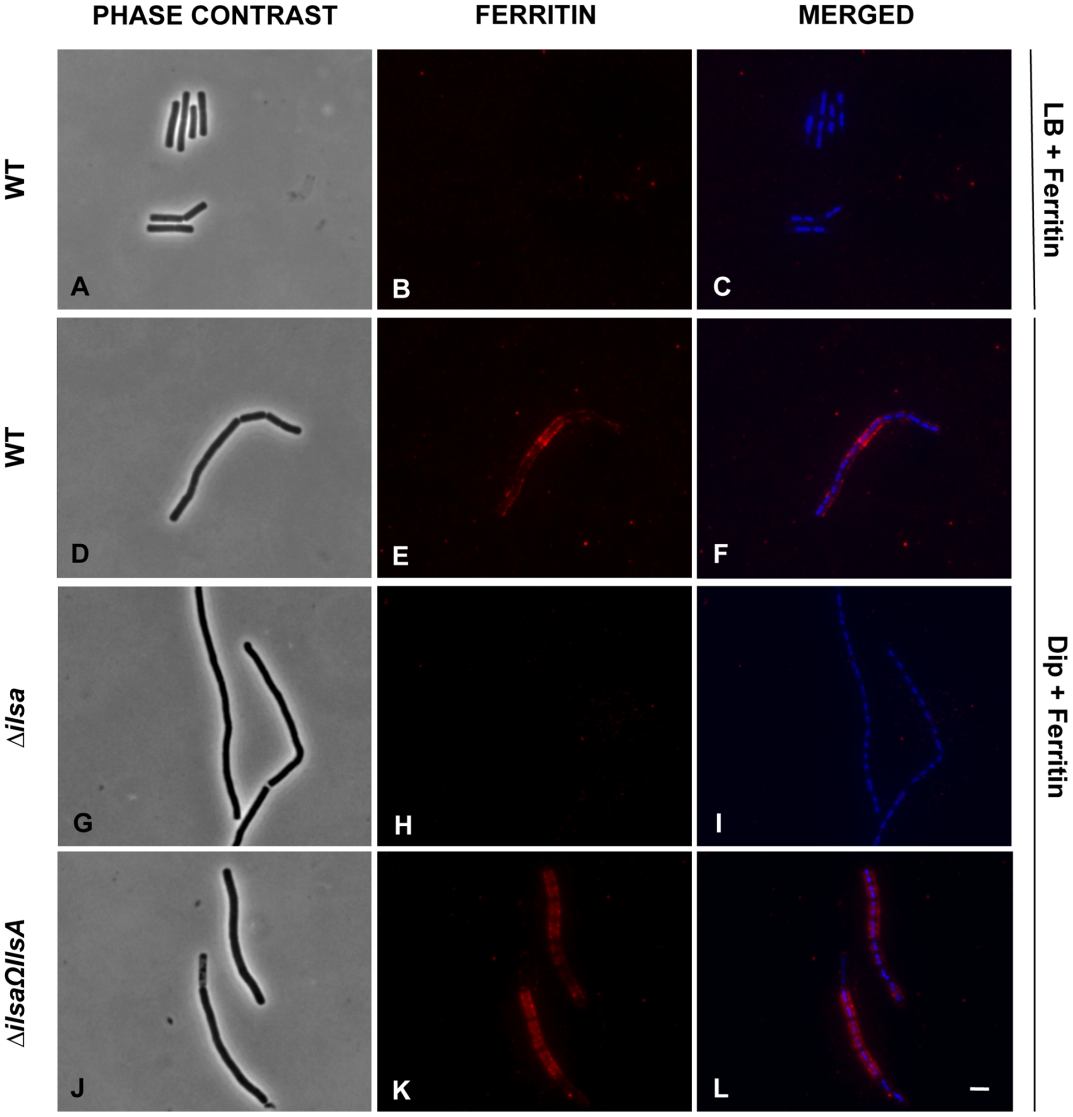
IlsA is essential for ferritin binding on *B. cereus* cell surface. *B. cereus* wild type (WT; A–F), *ΔilsA* mutant (G–I) and the complemented *ΔilsAΩilsA* (J–L) strains were grown in LB+0,3 µM HoSF (Horse Spleen Ferritin) medium (only the wild type; A–C) and in iron-depleted LB (Dip) +0,3 µM HoSF medium (D–L). Bacterial cells were washed before fluorescence microscopy analysis using HoSF Alexa Fluor 594 labelled polyclonal antibody (B,E,H,K) or DAPI to stain DNA. The merged images (C,F,I,L) show DAPI in blue and ferritin in red. Experiments were performed three times. In iron rich LB medium, IlsA is not produced [14] and no ferritin is detected on the bacterial surface in these conditions (A–C). Ferritin aggregates only on the surface in iron-depleted medium supplemented with ferritin (D–F). Absence of llsA in the Δ*ilsA* mutant compromises ferritin binding on the bacterial surface (G–I) whereas *ilsA* complementation restores ferritin aggregation (J–L).

Earlier studies showed that under iron-restricted conditions IlsA was located on the surface of *B. cereus*, and ELISA assays and Surface Plasmon Resonance measurements indicated a possible interaction between IlsA and ferritin *in vitro* [14]. To demonstrate the involvement of IlsA in the binding of ferritin to bacterial cells *in vivo*, ferritin localization was followed by immunofluorescence using the polyclonal antibody anti-HoSF (Horse Spleen Ferritin) (Figure 1). When the wild-type strain was grown in ferritin-enriched LB medium, a condition under which *ilsA* is not expressed [14], [33], ferritin was not immuno-detected on the bacterial cell surface. In sharp contrast, in iron-depleted medium supplemented with HoSF, ferritin aggregation was observed on the surface along the chains of bacterial cells. Moreover, ferritin recruitment was abolished in the *ΔilsA* mutant whereas complementation of this mutation with a wild-type copy of *ilsA* restored ferritin aggregation on the bacterial surface (Figure 1). Because *B. cereus* has its own bacterial ferritins, the antibodies were tested against the bacteria and no staining was observed in living cells; fluorescence was only detected in permeable dead cells (Figure S1). Collectively, our data are in agreement with the expression profile and the localization of IlsA during iron starvation [14], [33] and indicate that IlsA acts as a ferritin receptor *in vivo* (bacterial culture) too.

### IlsA interacts with each ferritin subunit

To further investigate the interaction of IlsA with the ferritin shell, the binding between the two purified proteins was followed *in vitro* by isothermal titration calorimetry (ITC). This highly sensitive thermodynamic technique measures heat variations during molecular interactions. In a single experiment, a complete thermodynamic profile of the interaction is obtained with the concomitant determination of the binding constant (*K*), the binding stoichiometry (*n*), the enthalpy (Δ*H*°), the entropy (Δ*S*°) and the free energy (Δ*G*°) changes of binding. The ITC technique has been proven very successful in obtaining accurate thermodynamic data for a number of molecular interactions involving ferritin [34], [35] or IlsA (Abi Khalil *et al.*, unpublished data). Each ITC experiment was repeated two to four times to ensure accuracy and reproducibility.

**Figure 2:**
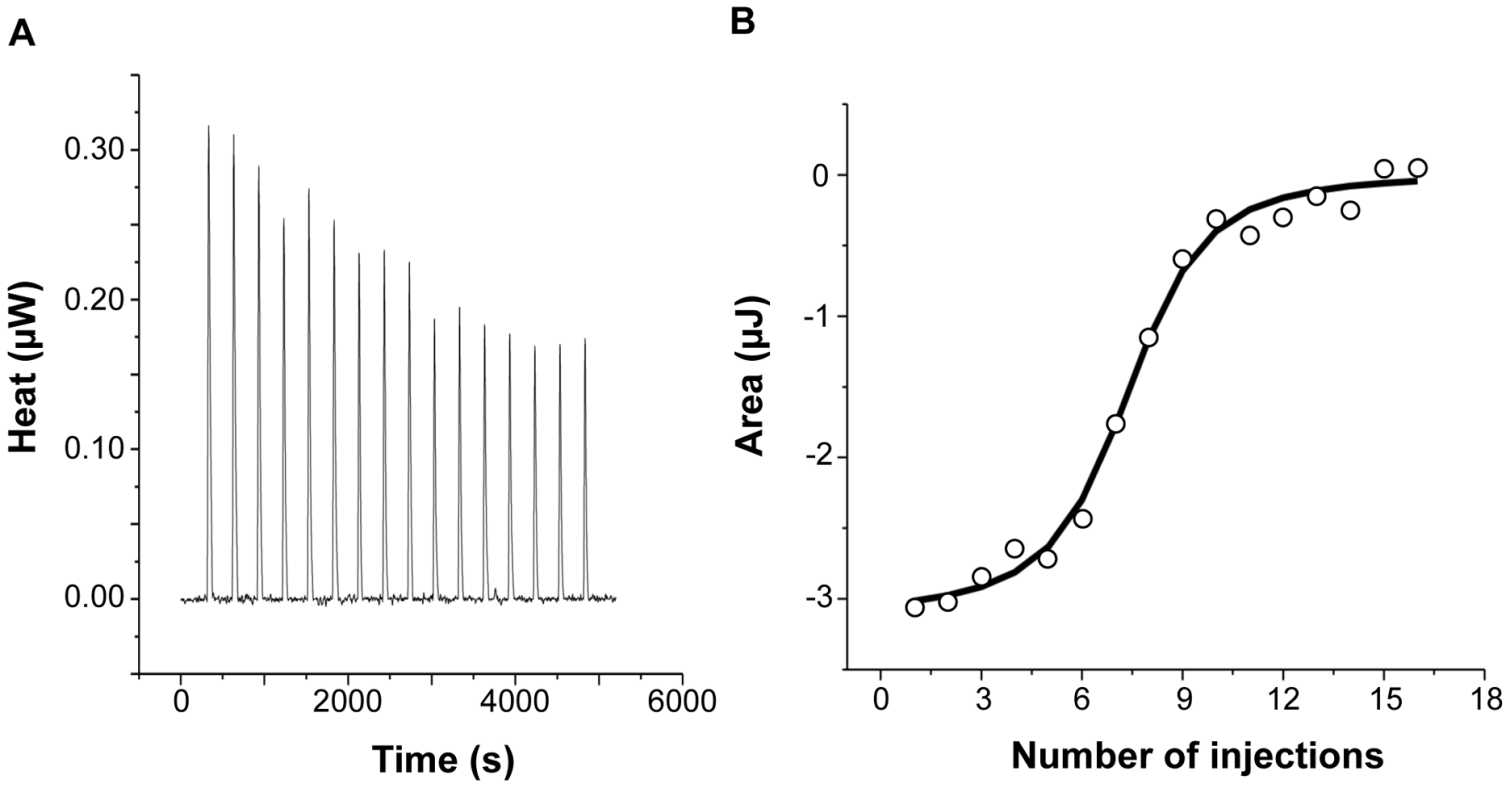
Calorimetric titration of recombinant human H-chain ferritin with IlsA. (**A**): ITC (Isothermal Titration Calorimetry) raw data. (**B**): Plot of the integrated heat versus the number of injections of IlsA. Conditions: 1 µM holoHuHF (recombinant Human H-chain Ferritin) titrated with 3 µl injections of 229 µM IlsA solution in 50 mM Tris/HCl buffer, 150 mM NaCl, 1 mM EDTA and 1 mM DTT, pH = 7.0 and 25°C. ITC binding experiments were repeated at least two times with similar results and thermodynamic data are listed in Table 1.

Figure 2 shows the injection heats for IlsA binding to recombinant human H-chain ferritin (HuHF) at pH 7.0 and 25°C (A) and the integrated heats (μJ) for each injection against the molar ratio of IlsA to HuHF after subtraction of the control heats (B). The other ferritins tested (recombinant human homopolymer L-chain, recombinant human heteropolymer ferritin composed of ∼20H-chains and 4L-chains and recombinant mouse homopolymer H-chain) showed similar ITC isotherms (Figure S2). All of the experimental thermodynamic parameters obtained from curve fittings of the integrated heats to a model of one set of independent binding sites are compiled in Table 1. As the concentration of IlsA increases following successive injections into the ITC reaction cell containing ferritin, saturation is reached and subsequently less heat is absorbed on further addition of IlsA. The positive upward peaks seen in Figure 2A correspond to an exothermic reaction with a binding stoichiometry of ∼24 IlsA per ferritin shell and an apparent dissociation constant (K_d_) of ∼540 nM. The binding of one IlsA molecule to one ferritin subunit did not alter binding of additional IlsA to the remaining subunits suggesting similar affinities and direct interactions between IlsA molecules and the 24-mer protein. The negative enthalpy change (∼−4 to −10 kJ/mol) and the large and positive entropy of binding (∼85 to 110 J/(mol.K)) observed with all IlsA-ferritin interactions indicate that the interaction is both enthalpically and entropically driven. The most likely contributions to the large positive ΔS^0^ values are probably due to changes in the hydration of the two proteins upon binding leading to an overall increase of the disorder of the system. To determine whether IlsA NEAT or LRR domains are involved in ferritin binding, dot blot experiments were performed separately on either domain following their individual expression and purification. No significant binding was observed between HoSF and the NEAT domain and HoSF was found to bind weakly to the LRR domains while a strong binding was apparent with full-length IlsA (Figure S3). These results suggest that the presence of both the NEAT and LRR domains may be crucial for optimal binding of ferritin to IlsA. However, we cannot exclude the possibility that the observed weak binding of HosF with the purified domains is a consequence of incorrect domain folding.

**Table 1:** Best fit parameters for ITC measurements of IlsA binding to ferritins^a^.

Protein 1	Protein 2	*n*	*K* (M^−1^)	Δ*H^0^* (kJ/mol)	Δ*G^0^* (kJ/mol)^b^	Δ*S^0^* (J/(mol.K))^c^
**Holo-IlsA**	**HuHF**	25.21±2.62	(1.86±0.99) ×10^6^	−4.11±0.11	−35.78±1.32	106.24±4.44
**Holo-IlsA**	**HuLF**	23.88±0.1	(8.36±0.24) ×10^5^	−8.71±3.15	−33.80±0.07	84.16±10.56
**Holo-IlsA**	**HuH/LF**	23.79±0.89	(1.08±0.21) ×10^6^	−8.72±2.47	−34.44±0.48	86.26±8.44
**Holo-IlsA**	**MoHF**	24.94±1.73	(2.07±0.66) ×10^6^	−3.35±0.32	−36.05±0.79	109.68±2.85

a The reported thermodynamic quantities are apparent values and include the contributions to the overall equilibrium from ferritin and buffer species in different states of protonation.

Standard errors from replicate determinations are indicated.

b Calculated from Δ*G* ^0^ = −*RT*ln*K*.

c Calculated from Δ*S* ^0^ = (Δ*H*°−Δ*G*°)/*T*.

HuHF, recombinant human H-chain ferritin; HuLF, recombinant human L-chain ferritin; HuH/LF, recombinant heteropolymer ferritin of 21H-chains and 4L-chains; MoHF, recombinant mouse H-chain ferritin. All experiments were repeated two to four times.

#### IlsA enhances iron release from ferritin

**Figure 3:**
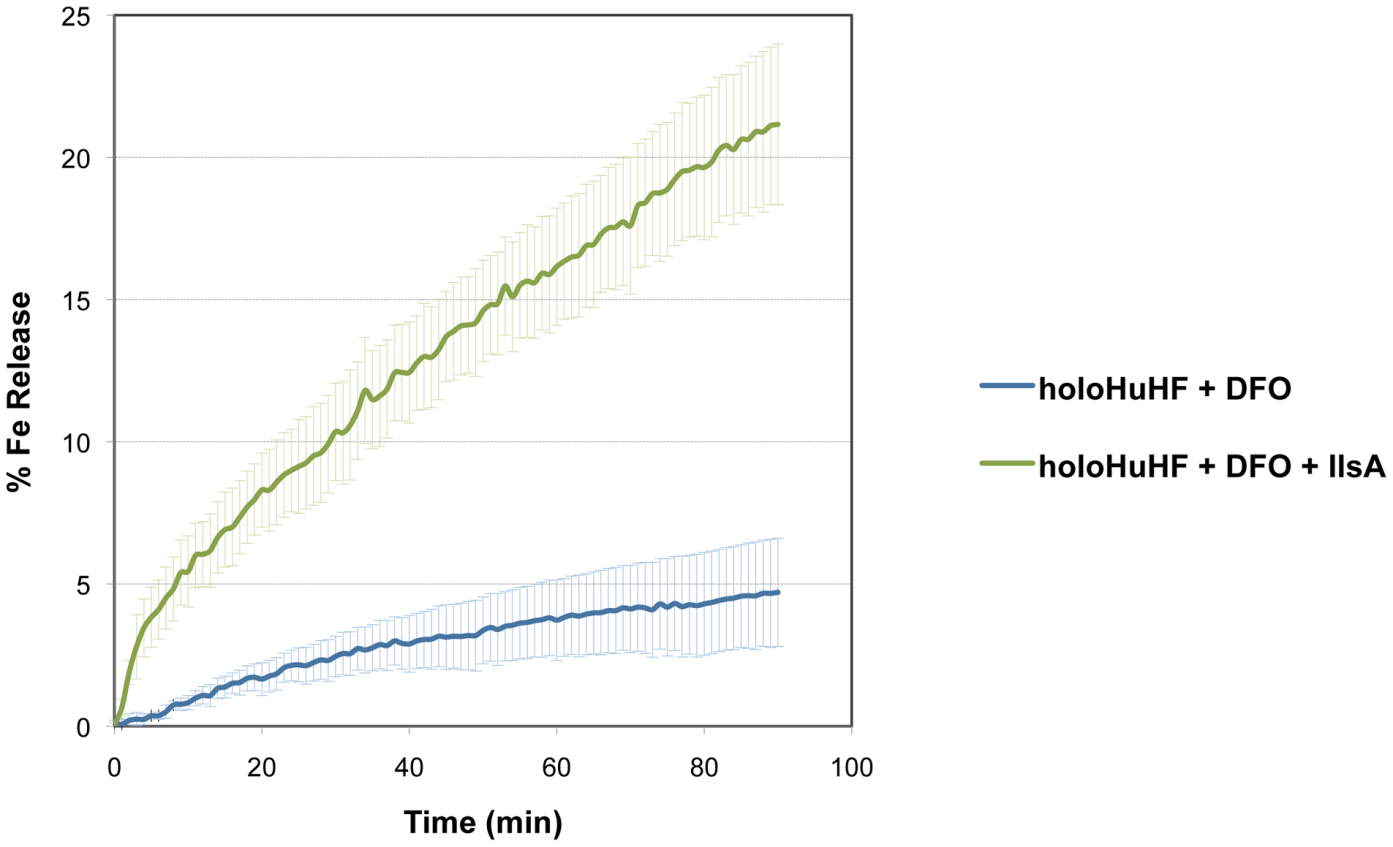
Role of IlsA in iron mobilization from ferritin. Demineralization of recombinant holoHuHF (recombinant Human H-chain Ferritin, 1 µM) containing 500 Fe/shell was followed by the absorption of the Fe(III)-DFO (Deferoxamine B) complex at 425 nm in the presence (black line) or absence (dotted line) of IlsA (5 µM). The experiment was repeated in triplicate using different protein preparations. Curves are averages of three independent runs and error bars are SEM from mean values.

To examine whether IlsA plays any role in iron mobilization from ferritin, *in vitro* spectrophotometric kinetic experiments using HuHF in the presence of IlsA were performed under aerobic non-reducing conditions. Because bacillibacftin is not available commercially and is very hard to purify, we used the bacterial siderophore DFO (Deferoxamine B) as a reporter molecule (i.e. an Fe(III)-chelator) to follow the kinetics of iron release from ferritin. Figure 3 shows that DFO alone (in the absence of IlsA) was able to release only a small amount of iron from HuHF loaded with ∼500 Fe/protein at a very slow rate (less than 5% after 90 minutes), a result in accord with earlier data obtained with other ferritins [36], [37], [38]. However, in the presence of llsA and DFO, a faster rate and a significant amount of iron was released from the protein (∼25% of total iron present within the protein) during the same time period (Figure 3) suggesting a role of IlsA in enhancing iron mobilization from ferritin. It is conceivable that IlsA might alter the ferritin structural integrity rendering the iron core more accessible to iron chelators such as microbial siderophores.

### Production of siderophores in *B. cereus*

**Figure 4:**
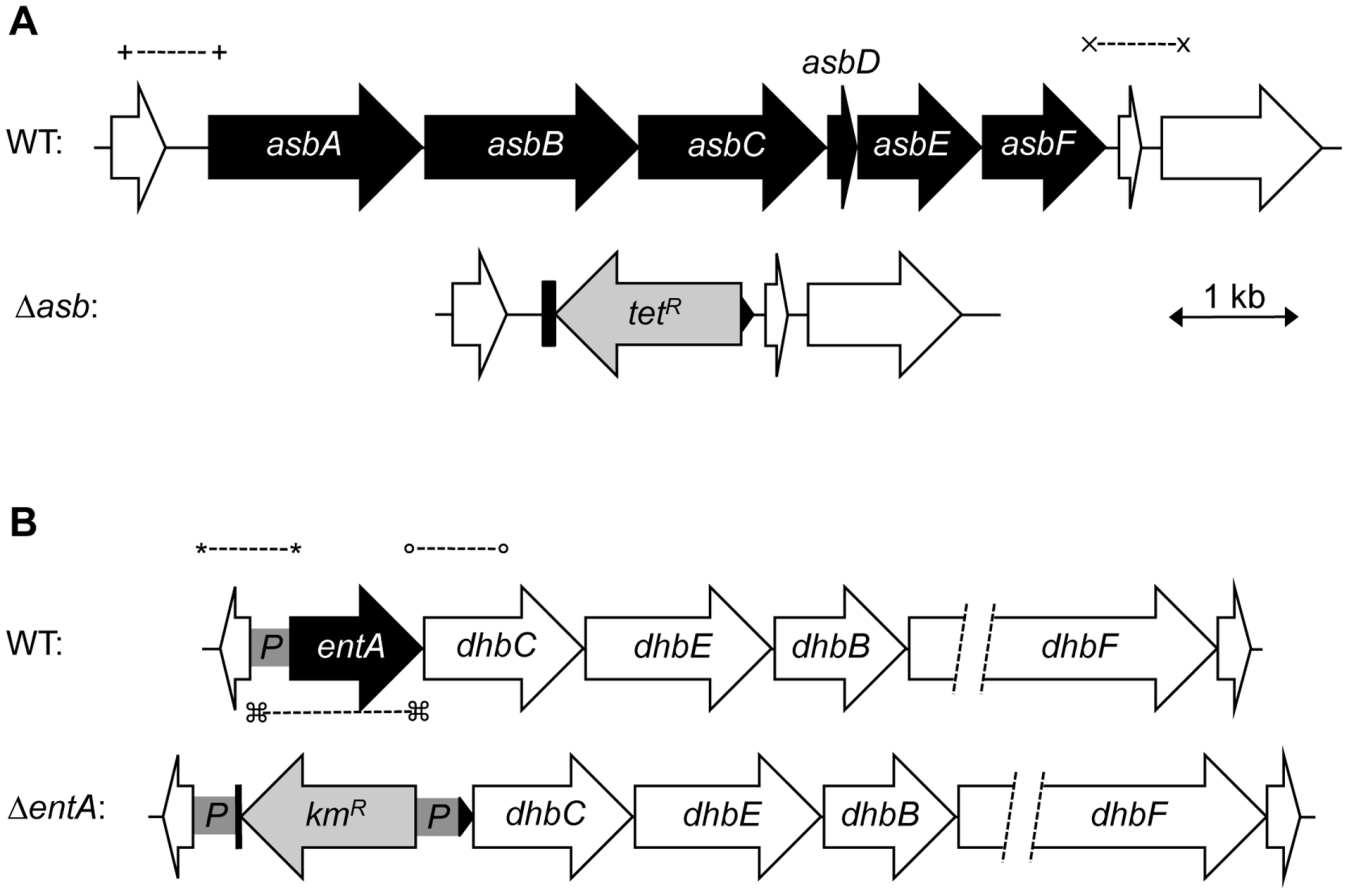
Construction of *B. cereus* siderophore mutants. Genetic organization of petrobactin (**A**) and bacillibactin (**B**) biosynthetic gene clusters in *B. cereus* strain ATCC 14579 is represented. The deleted genes (in black) and the orientation of the antibiotic cassettes (tetracycline, *tet^R^* and kanamycin, *km^R^*) are depicted. Deletions were created by integrative recombination in the loci using upstream and downstream region (∼1 kb each) amplified with primer pairs (+) and (x) for the *asb* locus or (*) and (°) for *entA* gene. In addition, the promoter region (359 bp) of *entA* was cloned between the *km^R^* cassette and *dhbC* (*P* in gray box) to ensure transcription of downstream genes. For *ΔentA* complementation, (□) represent the primers used to amplify the fragment cloned in pHT304 plasmid.

**Figure 5:**
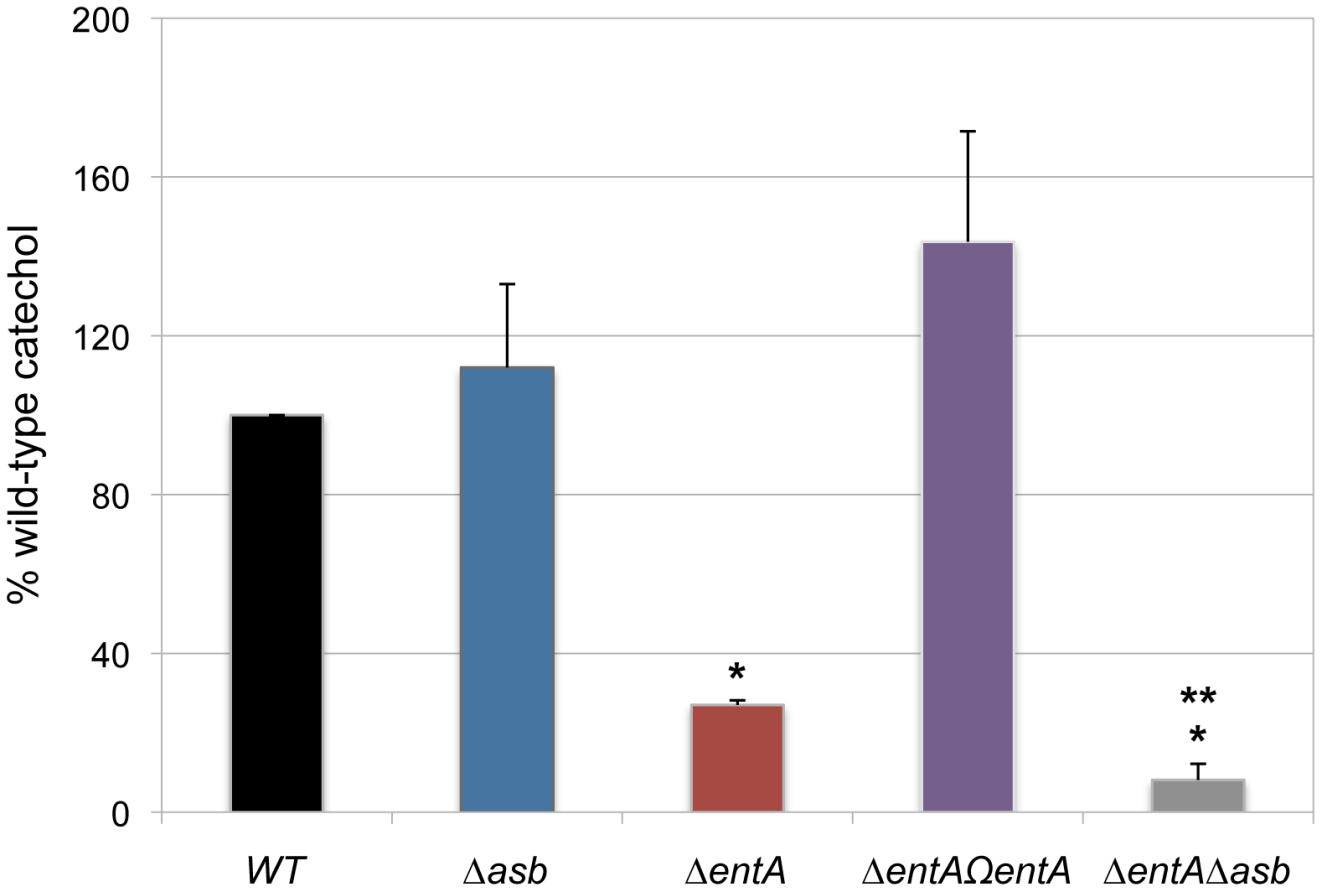
Production of catechol siderophores in *Δasb* and *ΔentA* mutants. Culture supernatants were collected for each strain from overnight (20 h) cultures in low iron conditions. Measurement of catechol productions was achieved using the Arnow assay. Data were normalized to the OD_600_ of cultures and percentages of wild-type (WT) catechol level are shown. Error bars represent SEM from mean values of three independent experiments. * Significant difference compared to wild type (*P*<0.001). ** Significant difference compared to *ΔentA* strain (*P*<0.05).

In iron-depleted medium, *B. cereus* and *B. anthracis* synthesize bacillibactin and petrobactin, two catechol-based siderophores that are differently regulated and have different affinity for iron [39], [40]. The organization of the biosynthetic gene clusters for both siderophores is highly conserved in the two species. Petrobactin and bacillibactin productions rely on the *asbABCDEF* and *entA-dhbBCF* clusters, respectively. Mutant strains were obtained from deletions of the *asbABCDEF* cluster and *entA* gene in the wild-type strain (Figure 4). To evaluate the relative contribution of the two siderophores in catechol production, the total siderophore production in wild-type and isogenic mutant strains *Δasb* and *ΔentA* and double mutant *ΔentAΔasb* were compared using the Arnow assay [41] (Figure 5). The expression of the *asbA* and *entA* genes was activated in the wild type strain grown under iron-depleted conditions (data not shown) and catechol production was detected in the wild type. The production of catechols was almost four times lower in the bacillibactin *ΔentA* mutant and the wild-type production was restored following complementation of *ΔentA* mutant with *entA* gene. In contrast, catechol production was not impaired in *Δasb* strain, suggesting a possible overproduction of bacillibactin to compensate for the absence of petrobactin. The strongest reduction in catechol production was observed in the *ΔentAΔasb* double mutant (Figure 5). These data indicate that bacillibactin represents the primary siderophore produced by *B. cereus* in low iron environment.

### Bacillibactin is essential for iron acquisition from ferritin

**Figure 6:**
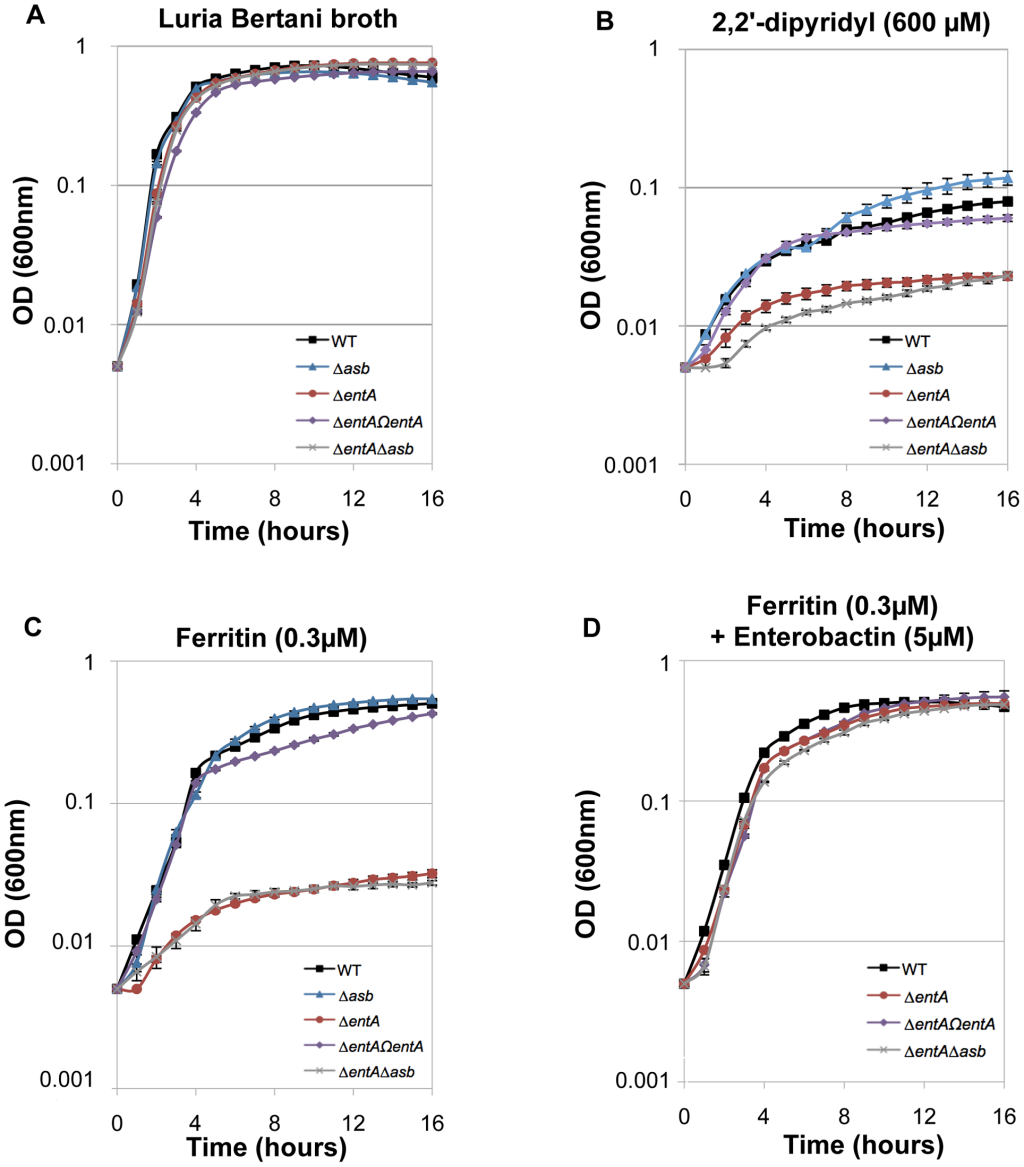
Iron acquisition from ferritin relies on bacillibactin production. Growth kinetics of *B. cereus* wild type (WT; black square), Δ*asb* petrobactin mutant (blue triangle), Δ*entA* bacillibactin mutant (red circle), complemented *ΔentAΩentA* strains (purple diamond) and double Δ*entA*Δ*asb* mutant (grey cross). The strains were grown at 37°C in LB medium (**A**) and in LB medium treated with 2,2′-dipyridyl without addition of iron sources (**B**) or supplemented with 0.3 µM ferritin only (**C**) or with 0.3 µM ferritin and 5 µM enterobactin (**D**). Bacterial growth was monitored during 16 hours by measuring the optical density (OD) at 600 nm every hour. Curves are averages of three independent experiments and error bars are SEM from mean values.

The ability of siderophores to remove iron from ferritin *in vitro* has been documented [42]. To investigate the ability of *B. cereus* siderophores to extract iron from ferritin *in vivo*, the growth of the wild type and mutant strains in different media was followed. No difference in growth was noticed in LB (Figure 6A). In iron-depleted medium, bacterial growth was strongly reduced for all strains (OD max after 16 h∼0.1), the *ΔentA* and *ΔentAΔasb* mutants being the most affected strains (Figure 6B). Supplementation with ferritin as sole iron source restored the growth of the wild type and the *Δasb* strains whereas the mutants disrupted in bacillibactin production were still unable to grow under these conditions (Figure 6C). The importance of *entA* (and therefore of bacillibactin) in iron acquisition from ferritin was further confirmed with the *ΔentΩentA* complemented strain since its ability to grow with ferritin was fully rescued (Figure 6C). Zawadzka *et al.* showed that the *B. cereus* FeuA transporter could bind both bacillibactin and the exogenous *E. coli* siderophore enterobactin [43]. Our data showed that the growth defect with ferritin due to the *entA* mutation was recovered when *E. coli* enterobactin was added to the culture medium (Figure 6D) suggesting that the FeuA transporter might be involved in the import process of bacillibactin too. The results of our study emphasize an important role of bacillibactin in iron acquisition from ferritin in *B. cereus* in contrast to previous work with *B. anthracis* pointing out a major role of the siderophore petrobactin in bacterial growth under iron starvation [44], [45], [46], [47].

### Bacillibactin is important for bacterial virulence in an insect

**Figure 7:**
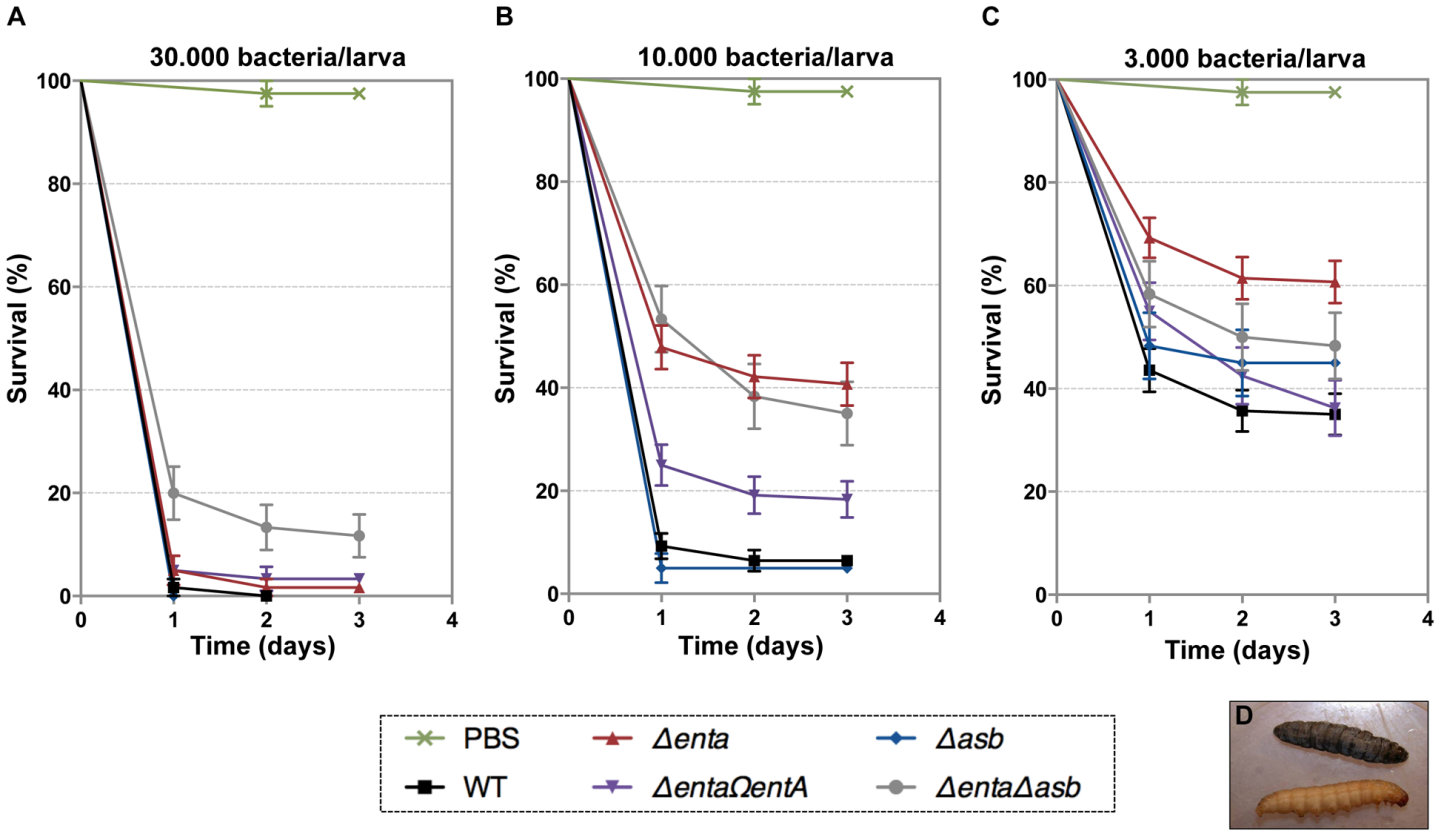
Effects of siderophore deficiency on *B. cereus* virulence in *G. mellonella* are dose- and time-dependent. Wild type and mutant strains were injected separately into the hemocoel. For each strain, twenty last-instar larvae were infected with 3×10^4^ (**A**), 1×10^4^ (**B**) or 3×10^3^ (**C**) of mid-log phase bacteria. The survival rate (% of alive/total number of infected larvae) was monitored for 72 hours after infection with the wild type (black square), Δ*asb* (blue diamond), Δ*entA* (red triangle), *ΔentAΩentA* (purple triangle), Δ*entA*Δ*asb* (grey circle) strains or PBS (green cross). Results are mean values of three to seven independent experiments and error bars indicate SEM from mean values. Based on these data, LD_50_ were estimated and are reported in Table 2. (**D**) Illustrates white alive and dead black larvae.

In addition to iron storage, ferritin serves as iron transporter in insects. High amounts of ferritin are found in the insect hemocoel (mg/L quantity) compared to the low level of vertebrate plasma ferritin (µg/L quantity) [4], [48]. Thus, insect ferritins represent an easily accessible iron-concentrated source for extracellular pathogens such as *B. cereus* and have been purified from the tissues and the hemolymph of the greater wax moth *Galleria mellonella* [49], [50], a very useful model to study bacterial pathogenesis [51]. While earlier studies have reported on the importance of IlsA in growth and survival of *B. cereus* in *G. mellonella* [14], [33], our current data suggest that bacillibactin acts in unison with IlsA in ferritin utilization. To investigate whether *B. cereus* siderophores are also involved in bacterial pathogenicity, virulence of the siderophore mutants was assayed in *G. mellonella* by direct injection into the hemocoel of various doses of mid-log phase bacteria (1×10^3^ to 3×10^4^). The number of living larvae was recorded for three days (Figure 7) and the 50% lethal doses (LD_50_) 24 hours after infection were evaluated by Probit analysis (Table 2). *ΔentA* and *ΔentAΔasb* mutants were significantly less virulent than the wild-type strain (Table S2) with a 5.6-fold and 6.7-fold decrease, respectively. At the highest dose, only the double mutant was affected. The virulence of the *ΔentA* strain complemented with wild-type *entA* (*ΔentAΩentA*) was partially restored and no difference was observed between the *Δasb* mutant and the wild-type strain. While most of the larvae died 24 hours after the injection of the wild type or the *Δasb* mutant, survival with the *ΔentA* mutant continued to decrease after the first 24 hours (Figure 7). Since petrobactin is the first siderophore produced upon bacterial outgrowth from spores in *B. anthracis* [47], the role of petrobactin in *B. cereus* virulence following inoculation with spores was then investigated. The infection tests with wild type and mutant spores yielded the same results as the previous assays with vegetative cells (data not shown). These results confirm that, in an insect model, bacillibactin plays a more important role than petrobactin in *B. cereus* virulence. Our data indicate that the strains impaired in bacillibactin production are still able to kill their host but slower than the wild type suggesting that bacillibactin is an important adaptation factor that allows *B. cereus* to disseminate into the low iron environment encountered in the insect hemocoel.

**Table 2:** Virulence of siderophore mutants in *G. mellonella.*

Strain	LD_50_ a	LD_50_ Confidence Limits
Wild type BcATCC14579	1.8×10^3^	3.1×10^2^–3.3×10^3^
*Δasb*	3.4×10^3^	1.2×10^3^–5.6×10^3^
*ΔentA*	1.0×10^4^	8.8×10^3^–1.2×10^4^
*ΔentAΩentA*	6.4×10^3^	4.7×10^3^–8.2×10^3^
*ΔentAΔasb*	1.2×10^4^	8.5×10^3^–1.6×10^4^

a The 50% lethal doses, in number of colony forming units (cfu), were evaluated by Probit survival.

analysis (*p*<0.05).

## Discussion

For pathogens, the ability to cope with the low iron environment encountered in the host is essential for tissue colonization. Thus, the production of efficient iron acquisition systems represents key factors. Because ferritin is the major iron storage protein found in living organisms, pathogens have developed efficient mechanisms to use this iron source and gain rapid access to sufficient quantities of iron. However, studies of the microbial determinants involved in host ferritin iron theft remain scarce. The present study showed that the bacterial surface protein IlsA interacts directly with the ferritin shell perhaps altering its structural integrity and leading to an amplification of iron release from the nanocage.

In an earlier work [14], an *in vitro* interaction between recombinant IlsA and horse spleen ferritin was reported. Here, a more detailed *in vivo* characterization of this interaction demonstrates that IlsA is required for ferritin recognition and recruitment at the bacterial surface. The observed binding stoichiometry of 24 IlsA per ferritin molecule argues in favor of a direct interaction of one IlsA molecule per ferritin subunit, irrespective of the ferritin source or type (Table 1) suggesting a role for IlsA as ferritin receptor. The similarities between the thermodynamic parameters reported in Table 1 using different ferritin types (i.e. homopolymers composed of 24 H-subunits or 24 L-subunits and heteropolymers composed of 21 H- and 4 L-subunits) suggest that either ferritin subunit binds IlsA equally well. This novel finding advances our understanding of iron acquisition by pathogens since to our knowledge no host-ferritin receptor has been identified thus far in microorganisms. The only existing clue was described in the pathogenic fungus *C. albicans*, where Als3, which has no structural homology with IlsA, was required for ferritin binding to hypha. However, the authors did not shown any direct interaction between Als3 and ferritin [13]. To further understand how IlsA interacts with ferritin and which domain(s) is involved in the binding, we searched for ferritin-binding receptors that share some degree of homology with IlsA. In mammals, several receptors associated with serum ferritin internalization have been described. Tim-2, a T cell immunoglobulin-domain and a mucin-domain protein expressed in various mouse tissues and TfR-1, the human transferrin receptor-1 are both able to recognize H-chain ferritin [52], [53] whereas Scara5, a class A scavenger receptor binds L-chain ferritin and is used to deliver iron to mouse kidney cells [54]. However, no homology or conserved domains exist or is evident between IlsA and these ferritin receptors.

IlsA has an original structure consisting of LRR and NEAT domains. LRR motifs are found in a broad range of proteins and are frequently involved in protein-protein interactions [55]. The NEAT domains are known to interact with heme and hemoproteins. However, the NEAT protein IsdA from *S. aureus* was shown to bind several non-heme host proteins [56]. A recent investigation from our laboratory showed that *B. cereus* IlsA NEAT domain alone exhibited affinity for heme binding (Abi Khalil *et al.*, unpublished data). Here, we tested individually the LRR and NEAT domains of *B cereus* IlsA for their ability to bind ferritin. Although a weak interaction was observed with the LRR domains alone but not with the NEAT domain, only the full length IlsA protein was able to effectively bind ferritin. However, we cannot exclude structural modifications in the LRR domains during purification, which would explain the weak binding affinity observed with these domains. Therefore, further in-depth structural and mutagenesis studies are needed to pinpoint the exact location of the binding site between IlsA and ferritin.

Among the NEAT proteins, only a few of them also carry LRR domains and have been found exclusively in the Firmicutes phylum. Besides IlsA, two other members of this composite NEAT protein family have been described in *S. pyogenes* (Shr) and in *B. anthracis* (Hal). Similarly to IlsA, both NEAT proteins are involved in heme acquisition and bacterial virulence but no role in ferritin iron acquisition has been reported yet [57], [58], [59], [60]. Interestingly, *B. anthracis* possesses an ORF encoding a protein termed BslL (*Ba1346*) [61] that is nearly identical to the last three fourths of IlsA with LRR domains followed by three SLH domains. BslK (Ba1093), another *B. anthracis* protein, shares some similarities with both IlsA NEAT and SLH domains [61]. BslK binds and directionally transfers heme to the Isd system [62]. However, the involvement of these proteins in ferritin iron acquisition has not been studied. Further experiments are needed to determine whether the ability of IlsA to bind ferritin is a universally conserved feature of the composite NEAT-LRR proteins found in other pathogenic bacteria.

IlsA-ferritin interaction is also shown to enhance the rate of iron release from ferritin. This result constitutes a major finding since no direct effect of a microbial protein on iron mobilization from host ferritin has ever been reported. As the supramolecular structure of the ferritin shell is extremely robust, it is possible that IlsA induces small conformational modifications upon binding leading to local destabilization of the ferritin subunits. This observation is in part supported by the large positive ΔS^0^ values obtained by ITC reflecting an increase in the system's disorder.

Several models for iron mobilization from the protein nanocage have been proposed but the exact *in vivo* mechanism of iron release remains poorly understood [63]. It has been suggested that iron ions exit through the eight gated-pores located at the 3-fold symmetry axis of ferritin [64]. Mutations of specific residues near the ferritin entry pores lead to an increase in iron release and are associated with localized unfolding without changes in the overall protein assembly [65]. Moreover, the 3-fold channels can be altered by chaotropic agents [66] and specific ferritin binding peptides [67]. By interfering with the protein intramolecular forces, these small molecules can significantly alter the rate of iron mobilization from ferritin, underlying the importance of pore flexibility in the transfer of iron in or out of the nanocage cavity. In addition to several ferritin receptors described above, a number of studies have reported the existence of other proteins that bind ferritin (for review, see [21]). In the absence of a clear mechanism of iron release from ferritin, it has been suggested that ferritin-binding proteins may cause opening or closing of the 3-fold channels and regulate iron storage or release. Hence, it is tempting to speculate that IlsA might act as a chaperone protein that dock around the 3-fold channels causing pore opening and rapid iron mobilization from ferritin. In contrast, the slow iron release rate observed with DFO alone (less than 5% of the total iron present in ferritin) is probably a consequence of the chelator size, which is too large to pass through the narrow ferritin pores. Direct chelation at the surface of the iron core has been reported with small bidentate Fe (III)-chelator [68], [69] and is unlikely to be relevant in our case. However, our proposed model of iron release involving IlsA contrasts with previous models for microbial iron acquisition from host ferritin. For instance, *B. cenocepacia* and *N. meningitidis* adopt direct or indirect proteolytic degradation strategy, respectively [17], [19]. Preliminary tests using protease inhibitors suggest that ferritin utilization in *B. cereus* does not rely on proteolysis *in vivo* (data not shown). Another strategy used by microbes is based on a reductase activity as proposed for *L. monocytogenes* and *C. albicans* [13], [22]. However, DFO can remove iron from ferritin in a non-reductive process [38] and our *in vitro* experiments were carried out under non-reducing conditions. Thus, it is likely that the reductive pathway is not relevant in *B. cereus* although enhancement of iron mobilization through redox processes is not excluded by the present study.

The effect of microbial siderophores on iron mobilization from ferritin has been emphasized thirty years ago [42]. Although two *B. cereus* siderophores are produced in iron-depleted media, our data suggest that only bacillibactin enables iron transfer from ferritin. Compared to earlier studies with the *ΔilsA* mutant [14], the *ΔentA* strain deficient in bacillibactin production displayed a more pronounced growth defect on ferritin. This indicates that bacillibactin is essential for iron uptake from ferritin and that IlsA may facilitate this process, as evidenced in the *in vitro* iron release kinetics (Figure 3). Furthermore, as the ferric iron core of ferritin is highly insoluble in aerobic conditions and neutral pH (K_sp_(Fe(OH)_3_) = 10^−39^) [63], the major influence of bacillibactin could be ascribed to its stronger affinity for ferric ions (K_f_ = 10^47.6^) [70] compared to petrobactin (K_f_ = 10^23^) [71]. Bacillibactin is not only important for growth with ferritin but also for bacterial virulence in an insect model. These results contrast with the major role played by petrobactin in *B. anthracis*. In this closely related species, petrobactin is the primary siderophore synthesized under conditions of iron starvation [45] and is important for virulence in mice and survival in macrophages [44]. Petrobactin, but not bacillibactin, possesses a unique ability to evade the mammalian siderophore scavenger protein named siderocalin [72]. It has been proposed that petrobactin is probably a trait required for pathogenesis in mammals [39]. Considering that no siderocalin homolog has been described in insects to date and that ferritin is more abundant in the insect hemocoel than in the vertebrate blood, the relevance of bacillibactin in *G. mellonella* is meaningful. However, depending on the host infected, the type of tissues and the available iron source, variation in the relative importance of the *B. cereus* siderophores might be expected. Further studies in mammal models are needed to elucidate this possibility.

**Figure 8:**
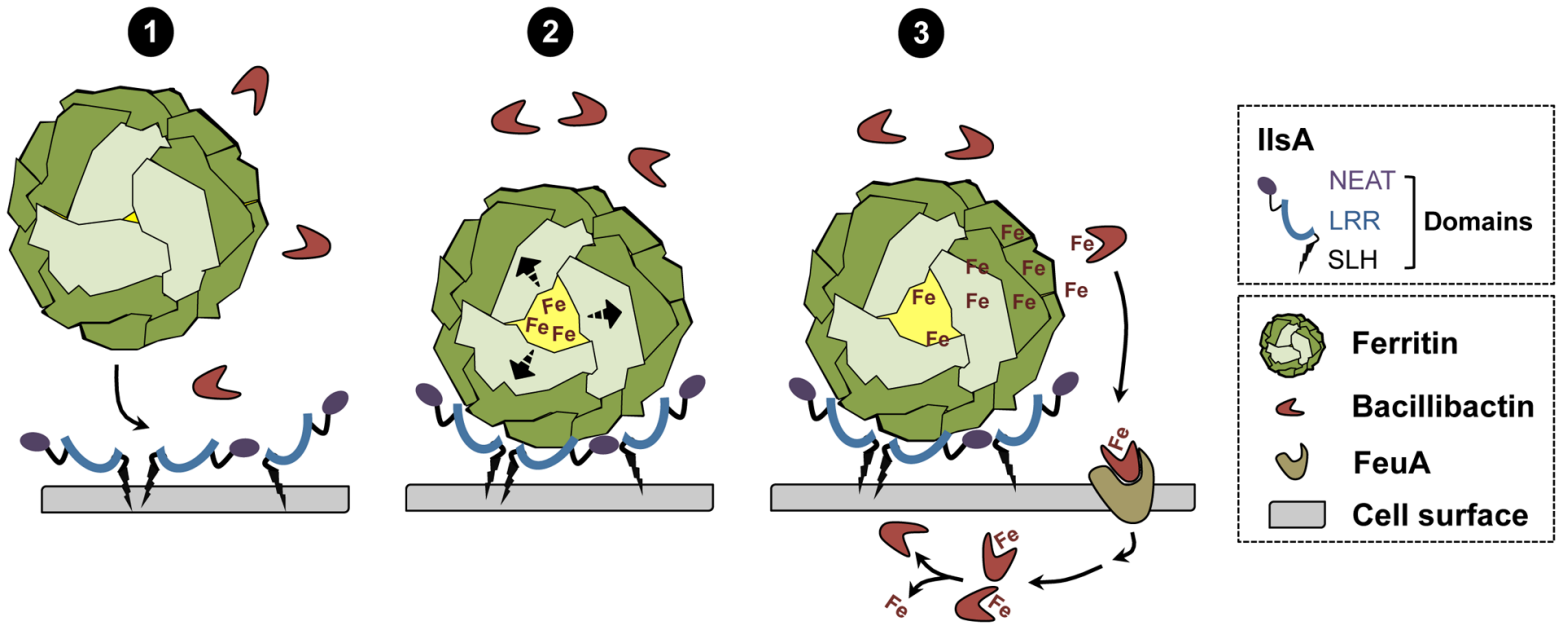
Schematic representation of iron uptake from ferritin in *B. cereus*. (1) In low iron environments, IlsA is produced and anchored to the surface. IlsA binds to each ferritin subunit, resulting in ferritin recognition and recruitment on the bacterial cell surface. (2) Following binding interaction, IlsA is proposed to alter ferritin pores openings or subunit-subunit interactions leading to protein destabilization or an increased accessibility to the ferritin iron core. (3) Because IlsA itself does not bind iron (data not shown), the iron released from ferritin by the action of IlsA is chelated by bacillibactin (and may involve other molecules) whereby the iron-siderophore complex is transported into the cell probably via the FeuA transporter leading to iron release into the cytosol.

In conclusion, a new model of iron mobilization from host ferritin in bacteria is proposed (Figure 8). This working model involves the destabilization of the ferritin subunits by IlsA leading to an enhancement of iron release from the ferritin mineral core. The *B. cereus* siderophore bacillibactin then acquires the mobilized iron needed for bacterial growth. The results of our current study and additional work from our laboratory [14] (Abi Khalil *et al.*, in revision) provide new insights into iron uptake by pathogens and ascribe a multifaceted role for IlsA in iron acquisition from structurally different host iron sources.

## Materials and Methods

### Bacterial strains and growth conditions

*Bacillus cereus* strain ATCC14579 (laboratory stock) was used throughout this study. The mutant *B. cereus* ATCC14579 *ΔilsA* was previously constructed by homologous recombination [33] and complemented with the pHT304Ω*ilsA* plasmid [14]. *E. coli* K12 strain TG1 was used as a host for cloning experiments. Dam^−^/Dcm^−^ *E. coli* strain ET12567 (laboratory stock) was used to generate unmethylated DNA for electro-transformation in *B. cereus*. *E. coli* strains M15 (laboratory stock) and C600 *ΔhemA* [73] were used for protein overproduction. All the strains used in this study are listed in Table 3. *E. coli* and *B. cereus* were cultured in LB (Luria-Bertani) broth, with vigorous shaking (175 rpm) at 37°C and *E. coli* C600 Δ*hemA* was cultured in BHI (Brain Heart Infusion, Difco) broth, without shaking. For electro-transformation, *B. cereus* was grown in BHI. *E. coli* and *B. cereus* strains were transformed by electroporation as previously described [74], [75]. The following concentrations of antibiotic were used for bacterial selection: ampicillin 100 µg ml^−1^ and kanamycin 25 µg ml^−1^ for *E. coli*; kanamycin 200 µg ml^−1^, tetracycline 10 µg ml^−1^ and erythromycin 10 µg ml^−1^ for *B. cereus*. The iron chelator, 2,2′-dipyridyl and the horse spleen ferritin (HoSF) were purchased from Sigma-Aldrich. 2,2′-dipyridyl was used at final concentrations of 200, 450 and 600 µM and ferritin at 300 nM.

**Table 3:** Strains and plasmids used in this work.

Strain or plasmid	Characteristics	Reference
**Strain**		
*Bacillus cereus* ATCC14579	Wild type	Laboratory stock
*Bc ΔilsA*	ATCC14579 mutant; *Δbc1331*; Tet^R^	[33]
*Bc ΔilsAΩilsA*	*ΔilsA* strain carrying pHT304Ω*ilsA* plasmid; Tet^R^, Erm^R^	[14]
*Bc Δasb*	ATCC14579 mutant; *Δbc1978–1983*; Tet^R^	This study
*Bc ΔentA*	ATCC14579 mutant; *Δbc2302*; Kan^R^	This study
*Bc ΔentAΩentA*	*ΔentA* strain carrying pHT304Ω*entA* plasmid; Kan^R^, Erm^R^	This study
*Bc ΔentAΔasb*	*Δasb* mutation into *ΔentA* strain; Kan^R^, Tet^R^	This study
*Escherichia coli* K12 strain TG1	Strain used as host for cloning experiments	Laboratory stock
*Ec* ET12567	Strain used for generation of unmethylated DNA	Laboratory stock
*Ec* C600 *ΔhemA*	Heme-deficient mutant used for protein overproduction; Kan^R^	[73]
*Ec* C600 *ΔhemA* GST-IlsA	Strain C600 *ΔhemA* carrying pGEX6P1-*ilsA*; Kan^R^, Amp^R^	This study
*Ec* C600 *ΔhemA* GST-NEAT^IlsA^	Strain C600 *ΔhemA* carrying pGEX6P1-*NEAT^IlsA^*, Kan^R^, Amp^R^	This study
*Ec* M15	Strain carrying pREP4, used for protein overproduction; Km^R^	Laboratory stock
*Ec* M15 GST-LRR^IlsA^	Strain M15 carrying pREP4 and pGEX6P1-*LRR^IlsA^*; Kan^R^, Amp^R^	This study
**Plasmid**		
pHT304	Shuttle vector used for complementation; Amp^R^, Erm^R^	[80]
pMAD	Shuttle vector, thermosensitive origin of replication; Amp^R^, Erm^R^	[78]
pRN5101	Shuttle vector, thermosensitive origin of replication; Amp^R^, Erm^R^	[83]
pHTS1	Vector carrying the tetracycline resistance cassette (*tet*)	[81]
pDG783	Vector carrying the kanamycin resistance cassette (*aphA3*)	[82]
pGEX6P1	Vector for inducible GST-tagged protein overproduction; Amp^R^	GE Healthcare
pMAD*Ωasb*::*tet*	pMAD with *bc1978–1983* deletion fragment	This study
pRN5101*ΩentA*::*kan*	pRN5101 with *bc2302* deletion fragment	This study
pHT304*ΩentA*	pHT304 with wild-type *entA* fragment	This study
pGEX6P1-*ilsA*	pGEX6P1 with the whole i*lsA* sequence (without signal peptide)	This study
pGEX6P1-*NEAT^IlsA^*	pGEX6P1 with *NEAT* domain sequence of *ilsA*	This study
pGEX6P1-*LRR^IlsA^*	pGEX6P1 with *LRR* domains sequence of *ilsA*	This study

*Bc*, *B. cereus*; *Ec*, *E. coli*; Tet, tetracycline; Erm, erythromycin; Kan, kanamycin; Amp, ampicillin.

### Immunofluorescence analysis

*B. cereus* wild type, *ΔilsA* and *ΔilsAΩilsA* strains were grown overnight in LB medium supplemented with 200 µM 2,2′-dipyridyl. These cultures were used to inoculate several media (LB/LB+0.3 µM HoSF/LB+450 µM 2,2′-dipyridyl/LB+450 µM 2,2′-dipyridyl+0.3 µM HoSF) at 37°C and a final OD of 0.1. Mid-log phase bacteria were collected, washed twice in PBS buffer and used immediately. Bacterial cells (∼10^8^) were fixed with 4% formaldehyde dissolved in PBS on poly-L-Lysine slides (Labomoderne). After 20 min, bacteria were washed with PBS, blocked with 1% BSA and incubated for one hour at room temperature with an anti-HoSF polyclonal antibody (Sigma-Aldrich) labeled with Alexa Fluor 594-conjugate (Invitrogen) at a dilution of 1∶60 in 1% BSA. Then, bacteria were washed with PBS, fixed a second time with 4% formaldehyde and bacterial DNA was counterstained with (4′,6-diamidino-2-phenylindole) DAPI diluted at 1∶300 in 1% BSA. Finally, slides were rinsed with water, cover-slips were sticked with the antifading Polyvinyl alcohol mounting medium with 1,4-diazabicyclo[2.2.2]octane (DABCO) from Fluka and dried at 37°C for 30 min. At least two experiments in duplicates were examined by phase contrast and epifluoresence microscopy using a Zeiss Observer Z1 microscope and the Axiovision imaging software. A representative picture of each strain was selected.

### Overproduction and purification of IlsA and its NEAT and LRR domains

GST-IlsA, GST-NEAT^IlsA^ and GST-LRR^IlsA^ were purified as recombinant proteins from *E. coli* using the expression plasmids pGEX6P1-*ilsA*, pGEX6P1-*NEAT^IlsA^* and pGEX6P1-*LRR^IlsA^*. In order to purify recombinant IlsA protein and its NEAT and LRR domains, sequences corresponding to Ala29-Lys760, Thr24-Gly163 and Lys208-Asn492 respectively were amplified from *B. cereus* strain ATCC14579 with paired primers FIlsA/RIlsA, FNEAT/RNEAT and FLRR/RLRR respectively (Table 4) and cloned into the plasmid pGEX6P1 holding the tag GST (GE Healthcare) after digestion of the PCR products with EcoRI/XhoI. Recombinant *LRR^IlsA^* were overexpressed in *E. coli* M15 as previously described in [14], except that the bacterial culture was incubated for 3 h at 30°C and overnight at 15°C. IlsA and NEAT^IlsA^ were produced in apo-form ((without heme bound to the NEAT domain) by using *E. coli* strain C600 *ΔhemA* impaired in heme biosynthesis [73]. The recombinant apo-proteins were expressed in BHI (Brain Heart Infusion, Difco) supplemented with appropriate antibiotics and the cultures were grown into bottles, in static phase, at 37°C to an OD_600_ = 0.8–0.9. The same protocol used for LRR^IlsA^ purification is then followed. The Bulk GST Purification Module (GE Healthcare) was used as recommended by the manufacturer. GST tag was removed by eluting the proteins with the PreScission Protease (GE Healthcare). The purified proteins were concentrated by ultrafiltration and stored at −20°C. For apo-IlsA and apo-NEAT^IlsA^, in order to reconstitute holo-proteins, hemin (Sigma-Aldrich) was added to protein preparations until saturation of 80%.

**Table 4:** Primers used in this work.

Name	Sequence 5′-3′	Restriction site (underlined)
FIlsA	CGGAATTCGCATTAAAAGTTGAAGCAAATC	EcoRI
RIlsA	CCCTCGAGTTATTTCTTTATTGCATTATAC	XhoI
FNEAT IlsA	CCGGAATTCACTCCAGCATTAGCGGCA	EcoRI
RNEAT IlsA	CCCTCGAGACCTACAGTTGGATCTTTAAT	XhoI
FLRR	CCGGAATTCAAAGATTTAAATACACC	EcoRI
RLRR	CCCTCGAGTCAATTTTGGACATTAATATAA	XhoI
FpetU	GGAATTCGATAGTTGGAAAGCAACG	EcoRI
RpetU	CGGGATCCATACAAAGTAACGTTCTG	BamHI
FpetD	AACTGCAGAATGGTTTGGACATAATTC	PstI
RpetD	GCGTCGACCTTGAATCGCTCTACCG	SalI
FbacU	CCAAGCTTGGTATTTACTTCGTATGTGTAG	HindIII
RbacU	GCTCTAGAGCCTATGCCTTGTGCTGCA	XbaI
FbacD	CCCTCGAGGCACAACCTTCAGAAGTTGC	XhoI
RbacD	CGGGATCCCGCTTCACTATGAATAACTGAT	BamHI
FbacP	AACTGCAGAAGCATTGTAAATGAACGTATC	PstI
RbacP	CCCTCGAGGGTTTCTCTATCCTTTCACATA	XhoI
FbacComp	AACTGCAGCATTGTAAATGAACGTATC	PstI
RbacComp	GCTCTAGATTAAACTCCTAACGTAGC	XbaI

### Microcalorimetry

Isothermal titration calorimetry (ITC) experiments were performed at 25°C on a low volume (185 µl) NanoITC (TA Instruments). Titrant and sample solutions were made from the same stock buffer solution (50 mM Tris- HCl pH 7, 150 mM NaCl, 1 mM EDTA, and 1 mM DTT). IlsA and its purified domains were obtained as described above. Concerning the ferritin samples, recombinant mouse H-chain (MoHF), human H-chain (HuHF), human L-chain (HuLF) and human heteropolymer H/L (HuH/LF) were purified as previously described [76], [77]. To test for the interaction between IlsA (or its NEAT and LRR domains) and ferritins, an automated sequence of 16 injections, each of 3 µl titrant (229 µM holo-IlsA) into the sample cell containing 1 µM ferritin, was performed at intervals of 5 min to allow complete equilibration, with the equivalence point coming at the area midpoint of the titration. The protein solution was stirred at 250 rpm to ensure rapid mixing of the titrant upon injection. The area under the resulting peak following each injection is proportional to the heat of interaction, which is normalized by the concentration of the added titrant and corrected for the dilution heat using the buffer solution alone to give the molar binding enthalpy ΔH°. The data were collected automatically and analyzed using NanoAnalyze fitting program (TA Instruments). The standard enthalpy change (ΔH°), the binding constant (K), and the stoichiometry of binding (n) are determined by a single ITC experiment. From these values, the standard Gibbs free energy change (ΔG°), and standard entropy change (ΔS°) are calculated using the following equations: ΔG° = −RTlnK and TΔS° = ΔH°−ΔG° where R is the universal gas constant (1.9872 cal mol^−1^ K^−1^) and T is the temperature in Kelvin degrees. The dissociation constant is expressed as K_d_ = 1/K (in mol l^−1^). All experiments were repeated two to four times and control experiments (IlsA or ferritin alone in the buffer) did not show any significant heat changes.

### Iron release assays

Apoferritin (HuHF) was loaded aerobically with 500 Fe atoms/nanocage. Typically, the FeSO_4_ solution was prepared in pH 2 DI water and loaded into ferritin via ten additions of 50 Fe(II) per shell. The iron release experiments were conducted in 50 mM Tris-HCl pH 7 and 150 mM NaCl in presence of 1 µM ferritin, 1 mM deferoxamine B (DFO – Sigma-Aldrich) chelator and with or without purified IlsA at 5 µM. The kinetics of iron release were performed under aerobic conditions at 25°C and monitored by the increase in the characteristic MLCT absorption band of the Fe(III)-DFO complex (425 nm). The percent of iron release from ferritin was calculated using experimentally determined UV-Vis molar extinction coefficient of the Fe(III)-DFO complex at 425 nm (3500 M^−1^ cm^−1^). Experiments were repeated three times with different protein preparations.

### DNA manipulations and plasmid constructions

Chromosomal DNA was extracted from *B. cereus* cells with the Puregene Yeast/Bact. Kit B (QIAgen). Plasmid DNA was extracted from *E. coli* and *B. cereus* using QIAprep spin columns (QIAgen). For *B. cereus*, 5 mg ml^−1^ of lysozyme was added and cells were incubated at 37°C for 1 h. Restriction enzymes and T4 DNA ligase were used according to the manufacturer's instructions (New England Biolabs). Oligonucleotide primers (Table 4) were synthesized by Sigma-Proligo. PCRs were performed in an Applied Biosystem 2720 Thermak cycler (Applied Biosystem) with Phusion High-Fidelity or Taq DNA Polymerase (New England Biolabs). Amplified fragments were purified using the QIAquick PCR purification Kit (QIAgen). Digested DNA fragments were separated by electrophoresis on 0.8% agarose gels and extracted from gels using the QIAquick gel extraction Kit (QIAgen). Nucleotide sequences were determined by Beckman Coulter Genomics.

The thermosensitive plasmids pMAD [78] and pRN5101 [79] were used for homologous recombination. The low-copy-number plasmid pHT304 [80] was used for complementation experiments with wild-type *entA* gene under its own promoter. The vector pGEX6P1 (GE Healthcare) was used to overproduce Glutathione S-transferase (GST)-tagged protein under the control of a *tac* promoter. All the plasmids used in this study are reported in Table 3.

### Construction of the *B. cereus* mutant strains

*B. cereus* Δ*asb* and *ΔentA* were constructed as follows. For *asbABCDEF* (*bc1978–1983*) deletion, a 956 bp EcoRI/BamHI DNA fragment and a 985 bp PstI/SalI DNA fragment, corresponding to the chromosomal regions located immediately upstream and downstream from the *asb* locus, were generated by PCR, using *B. cereus* strain ATCC14579 chromosomal DNA as a template and oligonucleotide pairs FpetU–RpetU and FpetD–RpetD respectively (Table 4). A Tet cassette, conferring resistance to tetracycline, was purified from pHTS1 [81] as a 1.6 kb PstI/BamHI fragment carrying the *tet* gene from *B. cereus*. The amplified DNA fragments and the Tet^R^ cassette were digested with the appropriate enzymes and inserted between the EcoRI and SalI sites of the thermosensitive plasmid pMAD [78] by ligation using the T4 DNA ligase.

For *entA* (*bc2302*) deletion, a 996 bp HindIII/XbaI and a 957 bp XhoI/BamHI DNA regions upstream and downstream the *entA* gene, were respectively amplified by PCR, using chromosomal DNA of the ATCC14579 strain of *B. cereus* as template and FbacU/RbacU, FbacD/RbacD as primers (Table 4). In addition, a 359 bp PstI/XhoI DNA fragment corresponding to the putative regulatory region of *entA-dhbBCF* was amplified using the same template and the primer pair FbacP/RbacP (Table 4). A Kan^R^ cassette containing *aphA3* gene, conferring resistance to kanamycin, was purified from pDG783 [82] as a 1.5 kb PstI/XbaI. The amplified DNA fragments and the Kan^R^ cassette were digested with the appropriate enzymes and inserted between the HindIII and BamHI sites of the thermosensitive plasmid pRN5101 [83] as illustrated in Figure 4B.

The resulting plasmids pMADΩ*asb*::*tet* and pRN5101Ω*entA*::*kan* were produced in *E. coli*, and then used to transform *B. cereus* wild type strain by electroporation. Integrants resistant to tetracycline (for *Δasb*) or kanamycin (for *ΔentA*) and sensitive to erythromycin arose through a double cross-over event, in which the chromosomal wild-type copies of *asbABCEDF* and *entA* coding sequences were deleted and replaced by the Tet^R^ and Kan^R^ cassette respectively. The chromosomal allelic exchanges were checked by PCR, using appropriate primers and by sequencing the insertion sites.

The genetic complementation of the *ΔentA* mutant was carried out as follows. A 1142 bp DNA fragment corresponding to the *entA* gene and its putative promoter was amplified by PCR using the *B. cereus* ATCC14579 genomic DNA as a template and FbacComp/RbacComp as primers (Table 4). The PCR product was digested with PstI and XbaI restriction enzymes and inserted into the plasmid pHT304 [80]. The resulting plasmid (pHT304Ω*entA*) was amplified in *E. coli* and then introduced into the *ΔentA* mutant strain of *B. cereus* by electroporation.

### Measurement of catechol production

Extracellular levels of catechols were measured using the Arnow assay [41]. Bacteria were grown overnight (20 h) at 37°C in LB medium +200 µM 2,2′-dipyridyl. Then, samples of cultures were collected, centrifuged and filtered to remove bacteria. Samples were mixed sequentially with equal volumes of 0.5 N HCl, nitrite-molybdate reagent (10% sodium nitrite and 10% sodium molybdate), and 1 N NaOH. Positive reactions produce a red colour and absorbance was determined at 510 nm. Data were normalized to OD600 of the original culture and percentages of wild-type catechol level in culture supernatants are presented. Three independent replicates were statistically analyzed using the Student's T-test.

### Growth assays

*B. cereus* strains were grown overnight at 37°C under low iron conditions by inoculating strains in LB medium supplemented with 200 µM 2,2′-dipyridyl. Overnight cultures were inoculated into a new LB medium +200 µM 2,2′-dipyridyl at a final OD of 0.01. Bacteria from mid-log phase culture were washed twice in LB medium containing 600 µM 2,2′-dipyridyl, then inoculated to a final optical density (OD) of about 0.005 into LB medium or LB+600 µM 2,2′-dipyridyl +0.3 µM HoSF supplemented or not with 5 µM Enterobactin (Sigma-Aldrich). Stock solution of ferritin was prechelated in 2 mM 2,2′-dipyridyl for two hours in order to eliminate the free iron. *B. cereus* cells were grown at 37°C in 96-wells microtiter plate under continuous shaking. The OD was measured at 600 nm every hour over 16 hours using a TECAN Infinite M200 Microplate Reader (TECAN Group). The assays were repeated at least three times.

### Virulence assays

Bacterial strains were grown in LB medium and bacterial concentrations were monitored by optical density measurements and plating dilutions onto LB agar plates. *B. cereus* wild-type and mutant strains were injected separately into the hemocoel of *G. mellonella*. Insect eggs were incubated at 25°C and the larvae reared on beeswax and pollen (Naturalim). Last-instar larvae weighing about 200 mg were injected with 10 µl of mid-log phase bacteria (or spores) suspended in PBS, using the microinjector (Buckard Scientific) as previously described [84]. Various doses of bacteria (1×10^3^ to 3×10^4^ bacteria/larva) were used, and each experiment was repeated at least three times with 20 larvae. A control group of larvae was injected with PBS only and no effect was observed. The survival rate (% of alive/total number of infected larvae) was recorded during 72 hours after infection. Statistical analysis was performed using the Log-rank test. Based on the data obtained, LD_50_ were estimated by Probit analysis with StatPlus software (AnalystSoft).

## Supporting Information

Figure S1 **Immunofluorescence control observations with anti-HoSF on B. cereus.** *B. cereus* wild type (A–D) was grown in iron rich LB medium. B: HoSF Alexa Fluor 594 labelled polyclonal antibody. C: DAPI, D: merged images (anti-HoSF: red, DAPI: blue). *B. cereus* ferrtitin is revealed inside lysed (dead bacterial) cells only, compare with DAPI staining in panel C and also with Figure 1. Experiments were performed three times.
(TIF)

Figure S2 **Calorimetric titration of various recombinant ferritins with IlsA.** (**A, C, E**): ITC raw data. (**B, D, F**): Plot of the integrated heat versus the number of injections of IlsA. Conditions: 1 µM HuLF (Human L-chain Ferritin; A, B) or HuH/LF (Human heteropolymer H/L Ferritin; C, D) or MoHF (Mouse H-chain Ferritin; E, F) titrated with 3 µl injections of 229 µM IlsA solution in 50 mM Tris/HCl buffer, 150 mM NaCl, 1 mM EDTA and 1 mM DTT, pH = 7.0 and 25°C. ITC binding experiments were repeated at least two times with similar results and thermodynamic data are listed in Table 1.
(TIF)

Figure S3 **Roles of the IlsA-NEAT and LRR domains in ferritin binding.** Dot blot experiments were carried as follows: 12 pmol of IlsA and the NEAT and LRR domains of IlsA purified separately were spotted on PVDF membranes and then incubated for 1 hour with HoSF at 1 µg/ml. The signals were obtained with the HRP (horse radish peroxidase) ECL (enhanced chemiluminescent) system using an anti-HoSF polyclonal antibody at a dilution of 1∶1000 in TBS pH 7.4 buffer with 1% fat free milkpowder.
(TIF)

Table S1 **The table refers to** ***B.cereus*** **genes, which have been studied in relation to iron acquisition particularly with attention to genes analyzed in an insect environment.**
(DOC)

Table S2 ***Galleria mellonella*** **larvae were infected by injection of several doses of** ***B. cereus*** **wildtype (WT) and various mutant strains of the EntA (bacillibactin) and Asb (petrobactin) siderophores.** Controls were infected with PBS buffer only. For survival curves see Figure 7.
(DOC)
