# Supplementary figures and images for: Iron Acquisition in Bacillus cereus: The Roles of IlsA and Bacillibactin in Exogenous Ferritin Iron Mobilization
Source: PLoS Pathog. 2014 Feb 13;10(2):e1003935. doi: 10.1371/journal.ppat.1003935 (PMC3923779; doi:10.1371/journal.ppat.1003935)

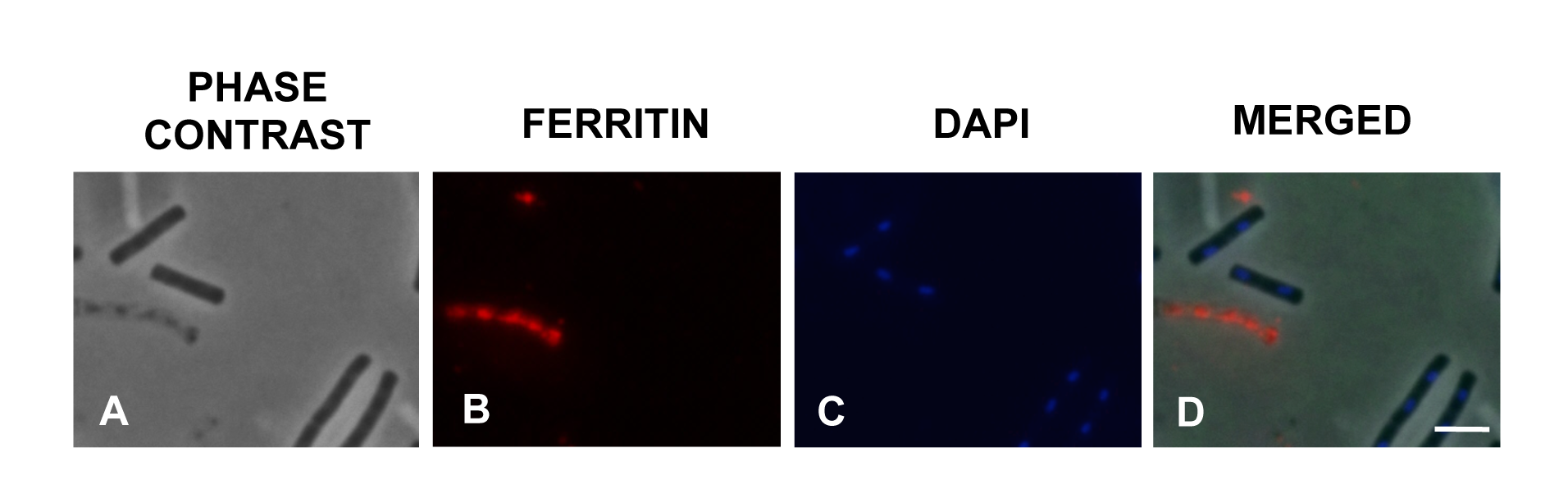

Supplement: Figure S1 — Immunofluorescence control observations with anti-HoSF on B. cereus. B. cereus wild type (A–D) was grown in iron rich LB medium. B: HoSF Alexa Fluor 594 labelled polyclonal antibody. C: DAPI, D: merged images (anti-HoSF: red, DAPI: blue). B. cereus ferrtitin is revealed inside lysed (dead bacterial) cells only, compare with DAPI staining in panel C and also with Figure 1. Experiments were performed three times. (TIF) [file ppat.1003935.s001.tif]

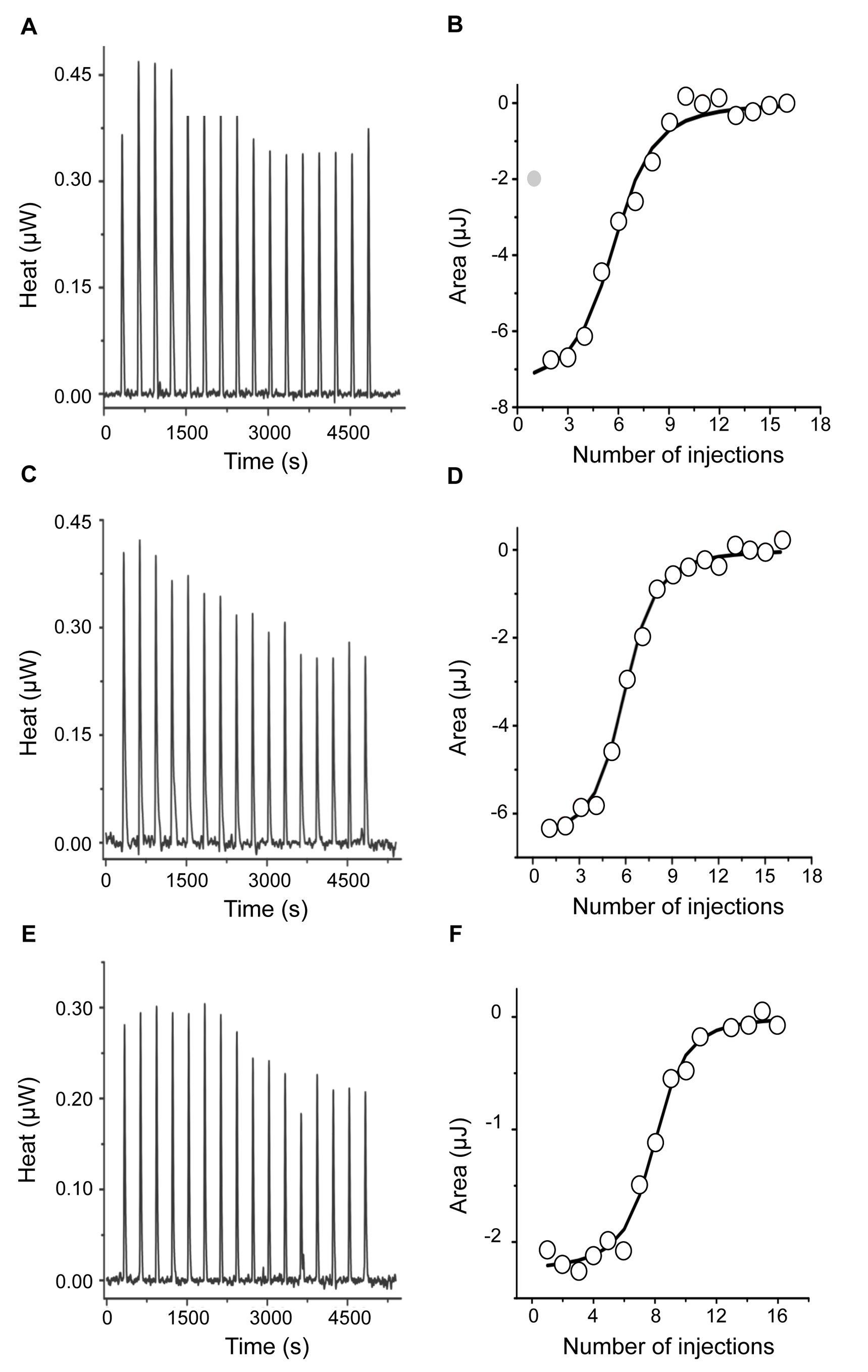

Supplement: Figure S2 — Calorimetric titration of various recombinant ferritins with IlsA. (A, C, E): ITC raw data. (B, D, F): Plot of the integrated heat versus the number of injections of IlsA. Conditions: 1 µM HuLF (Human L-chain Ferritin; A, B) or HuH/LF (Human heteropolymer H/L Ferritin; C, D) or MoHF (Mouse H-chain Ferritin; E, F) titrated with 3 µl injections of 229 µM IlsA solution in 50 mM Tris/HCl buffer, 150 mM NaCl, 1 mM EDTA and 1 mM DTT, pH = 7.0 and 25°C. ITC binding experiments were repeated at least two times with similar results and thermodynamic data are listed in Table 1. (TIF) [file ppat.1003935.s002.tif]

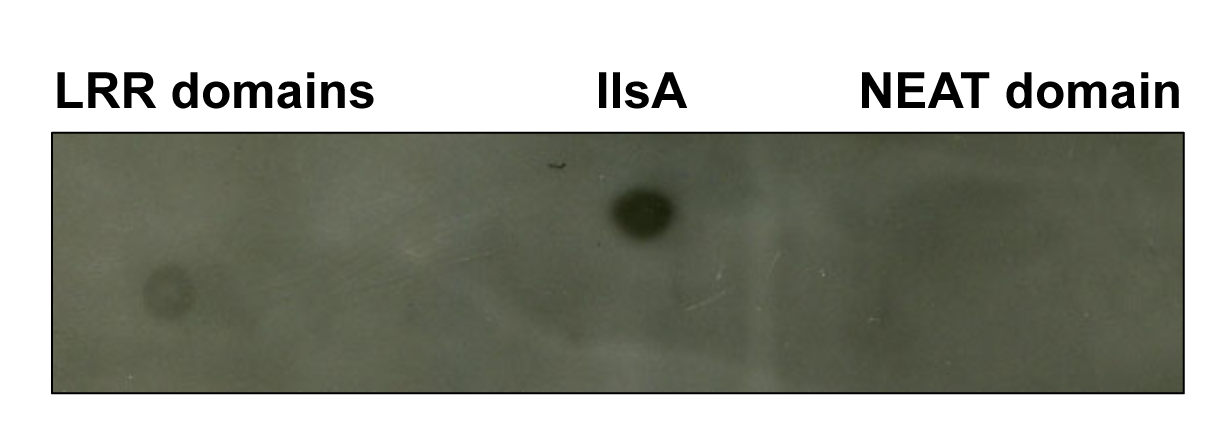

Supplement: Figure S3 — Roles of the IlsA-NEAT and LRR domains in ferritin binding. Dot blot experiments were carried as follows: 12 pmol of IlsA and the NEAT and LRR domains of IlsA purified separately were spotted on PVDF membranes and then incubated for 1 hour with HoSF at 1 µg/ml. The signals were obtained with the HRP (horse radish peroxidase) ECL (enhanced chemiluminescent) system using an anti-HoSF polyclonal antibody at a dilution of 1∶1000 in TBS pH 7.4 buffer with 1% fat free milkpowder. (TIF) [file ppat.1003935.s003.tif]
