# Supplementary material for: Iron Acquisition in Bacillus cereus: The Roles of IlsA and Bacillibactin in Exogenous Ferritin Iron Mobilization
Source: PLoS Pathog. 2014 Feb 13;10(2):e1003935. doi: 10.1371/journal.ppat.1003935 (PMC3923779; doi:10.1371/journal.ppat.1003935)
Supplement: Table S1 — The table refers to B.cereus genes, which have been studied in relation to iron acquisition particularly with attention to genes analyzed in an insect environment. (DOC) [file ppat.1003935.s004.doc]

**Table S1: Iron-related genes characterized so far in *B. cereus***

| **Gene name** | **Strain** |  | **Description** | **Role in virulence**  **(Model)** | **Reference** |
| --- | --- | --- | --- | --- | --- |
| *entA* | *Bc* ATCC14579 |  | BB biosynthesis | Yes (*Gm*) | This study |
| *feuA* | *Bc* ATCC14579 |  | BB and Ent transporter | nt | [43] |
| *asbABCDEF* | *Bc* ATCC14579 |  | PB biosynthesis | No (*Gm*) | This study |
| *fatB* | *Bc* ATCC14579 |  | PB and 3,4-DHB transporter | nt | [43] |
| *fpuA* | *Bc* ATCC14579 |  | PB and 3,4-DHB transporter | nt | [43] |
| *yfiY* | *Bc* ATCC14579 |  | Schizokinen transporter | nt | [43] |
| *yxeB* | *Bc* ATCC14579 |  | DFO and Ferrichrome transporter | nt | [43,] Fukushima *et al.* 2013 |
| *ilsA* | *Bc* ATCC14579 |  | Heme and ferritin receptor | Yes (*Gm*) | [14,33]; (Abi Khalil *et al.*, unpublished data); this study |
| *isd* locus | *Bc* ATCC14579 |  | Heme uptake system | No (*Gm*) | (Abi Khalil *et al.*, unpublished data) |
| *fctC* | *Bc* ATCC14579 |  | Ferric citrate complexes transporter | nt | [26] |
| *fecABC* | *Bc* 569 |  | Ferric dicitrate uptake system | Yes (*Ms*) | [27] |
| *Fur* | *Bc* 569 |  | Regulator | Yes (*Ms*) | Harvie *et al.* 2005 |
| *HlyII* | *Bt* 407 Cry^-^ |  | Pore-forming cytotoxin | Yes (*Mm* & *Bm*) | [32] Tran *et al.,* 2011 |

*Bc, Bacillus cereus. Bt, Bacillus thuringiensis. Gm, Galleria mellonella. Ms, Manduca sexta. Mm, Mus musculus. Bm, Bombyx mori.* nt, non tested.

References. **Fukushima** *et al.* (2013) Gram-positive siderophore-shuttle with iron-exchange from Fe-siderophore to apo-siderophore by Bacillus cereus YxeB. Proc Natl Acad Sci U S A 110: 13821-13826.**Harvie** *et al., (*2005) Bacillus cereus Fur regulates iron metabolism and is required for full virulence. Microbiology 151: 569-577.**Tran** *et al.* (2011) Haemolysin II is a Bacillus cereus virulence factor that induces apoptosis of macrophages. Cell Microbiol 13: 92-108.
