# Supplementary material for: Iron Acquisition in Bacillus cereus: The Roles of IlsA and Bacillibactin in Exogenous Ferritin Iron Mobilization
Source: PLoS Pathog. 2014 Feb 13;10(2):e1003935. doi: 10.1371/journal.ppat.1003935 (PMC3923779; doi:10.1371/journal.ppat.1003935)
Supplement: Table S2 — Galleria mellonella larvae were infected by injection of several doses of B. cereus wildtype (WT) and various mutant strains of the EntA (bacillibactin) and Asb (petrobactin) siderophores. Controls were infected with PBS buffer only. For survival curves see Figure 7. (DOC) [file ppat.1003935.s005.doc]

**Table S2: Statistical analysis of *Galleria mellonella* survival rates**

|  | **Bacteria/larva** | **PBS** | **WT** | ***∆asb*** | ***∆entA*** | ***∆entAΩentA*** | ***∆entA∆asb*** |
| --- | --- | --- | --- | --- | --- | --- | --- |
| **WT** | 3000 | ** | - | - | - | - | - |
|  | 10000 | ** | - | - | - | - | - |
|  | 30000 | ** | - | - | - | - | - |
|  |  |  |  |  |  |  |  |
| ***∆asb*** | 3000 | ** | ns | - | - | - | - |
|  | 10000 | ** | ns | - | - | - | - |
|  | 30000 | ** | ns | - | - | - | - |
|  |  |  |  |  |  |  |  |
| ***∆entA*** | 3000 | ** | ** | * | - | - | - |
|  | 10000 | ** | ** | ** | - | - | - |
|  | 30000 | ** | ns | ns | - | - | - |
|  |  |  |  |  |  |  |  |
| ***∆entAΩentA*** | 3000 | ** | ns | ns | ** | - | - |
|  | 10000 | ** | * | * | ** | - | - |
|  | 30000 | ** | ns | ns | ns | - | - |
|  |  |  |  |  |  |  |  |
| ***∆entA∆asb*** | 3000 | ** | * | ns | ns | ns | - |
|  | 10000 | ** | ** | ** | ns | ** | - |
|  | 30000 | ** | ** | ** | * | * | - |

*p* values were calculated using the Log-rank test. * (*p* < 0.05) and ** (*p* < 0.005), significant differences; ns, not significantly different.
